# Supplementary material for: Study on ring-road incident duration based on latent class accelerated hazard model
Source: PLoS One. 2024 Aug 12;19(8):e0308473. doi: 10.1371/journal.pone.0308473 (PMC11318924; doi:10.1371/journal.pone.0308473)
Supplement: Attachment — Submitted filename: Response to Reviewers.docx [file pone.0308473.s001.docx]

Dear Editor and Reviewers:

Thank you for your letter and for the reviewers' comments concerning our manuscript entitled " Study on ring-road incident duration based on latent class accelerated hazard model " (ID: PONE-D-24-18829). Those comments are all valuable and very helpful for revising and improving our paper, as well as the important guiding significance to our research. We have carefully studied the comments and made corrections, which we hope can meet with approval. These revised and added portions are marked in red on the paper. The main corrections in the paper and the responds to the reviewer's comments are listed bellow.

We tried our best to improve the manuscript and made some changes to this revised manuscript. However, these changes will not influence the content and framework of the paper.

We appreciate for Editors/Reviewers' warm work earnestly and hope that the correction will meet with approval.

Once again, thank you very much for your comments and suggestions.

Best regards,

Gen Li et al.

Response to the Reviewer's Comments:

Reviewer #1

Reviewer #1: Comment #1: The introduction of the article summarizes the contributions made in the field of incident duration research in the past. However, some recent studies on freeway safety could add value to the analysis of incidents.

*Authors' Response:* Thank you for your comments. We have discussed the latest research in depth and further combined it with this paper to add credibility and application value to this paper.

Reviewer #1: Comment #2: How are the characteristic classifications of each variable in Table 1 obtained? If they are extracted from other articles, proper references should be provided.

*Authors' Response:* Before analyzing the data, we first categorize it. The explanatory variables are divided into incident characteristics, temporal characteristics, environmental characteristics, traffic characteristics and operational characteristics, this classification method is quoted in other literatures, which can describe the data content more clearly and further improve the scientific nature of the paper. We have added the corresponding literature citations in "Data".

Reviewer #1: Comment #3: Line 140: What is the purpose of the Spearman correlation test? What does an absolute correlation coefficient of less than 0.7 indicate? This should be explained in the text.

*Authors' Response:* We have explained the purpose of Spearman correlation test in the "Data" of the revised manuscript. This is to verify that there is no multicollinearity in the optimal combination of variables after stepwise regression. At the same time, absolute correlation coefficient of less than 0.7 further proves that there is no close correlation in the combination of variables, excluding the possible influence of the additive effect of other factors.The details are as follows:

*To further verify whether there is multicollinearity and tight correlation in the variable combinations after stepwise regression, the Spearman correlation test was conducted to assess the relationship between the aforementioned variables. The results revealed that the absolute values of the correlation coefficients were less than 0.7, as displayed in Table 2. This shows that there is no tight correlation in the optimal combination of variables obtained by stepwise regression, further excluding the additive effects of other factors.*

Reviewer #1: Comment #4: The conclusion of the manuscript is too extensive and repeats the content of the discussion. A brief overview of the manuscript's content and its contributions would suffice.

*Authors' Response:* Thank you for your comments. We have further simplified the content of the "Conclusion", removing the unimportant content that repeats with the "Discussion", so as to simply describe the content and contribution of the manuscript. We believe this will increase the scientific nature of the article.

Reviewer #2

Reviewer #2: Comment #1: L27-28: Semantic relationship of some sentences in the abstract could not be established, there may be some grammar errors. Careful sentence review is very important.

*Authors' Response:* Semantic relationship of some sentences in the abstract has been optimized. At the same time, we have further reviewed the sentences and grammar of the paper in order to increase the credibility and scientific nature of the paper.

Reviewer #2: Comment #2: L125: The fonts and lines provided in Figure 1 do not seem to be rigorous enough. Please review them carefully.

*Authors' Response:* Thank you for your comment. The lines and fonts in Figure 1 were optimized to ensure that it can clearly and effectively reflect the overall idea and process of data processing.

Reviewer #2: Comment #3: As a special freeway, the ring-road has some differences from the general freeway, which need to be explained. In addition, the existing research in the field is based on the freeway, and whether there will be significant changes in the research methods for the ring-road is worth further exploration.

*Authors' Response:* In the penultimate paragraph of the introduction, we show the characteristics and functions of the ring-road, which are different from general freeways. In the meanwhile, it has a better supporting role for the research of this paper. Of course, because the ring road is a special freeway, there are some similarities in the research ideas. The method of this study is inspired by the previous research on the duration of freeway incidents, and the choice is further thought. Further discussion is significant on the need to find specific research methods for the ring-road. This is also one of the directions that we can study in the future. If there is a chance, we hope to continue in-depth and get guidance from experts. The specific contents are as follows:

*As a special freeway in China, the ring-road is mainly used to relieve the traffic pressure in urban areas, divert vehicles, and build a three-dimensional freeway network by forming an interchange with various high-grade freeways leading to the main urban area. The ring-road has some characteristics besides the general freeway, such as weak tidal property, many entrances and exits, complex sections, etc.*

Reviewer #2: Comment #4: The meaning of the Data section seems to be unclear. What is the purpose of describing the proportion of each type of incident? Does it relate to the main point or innovation of the article?

*Authors' Response:* Thank you for your comment. The Data section is mainly used to introduce the overall situation of the data, so that the data support of the article can be described more scientifically and clearly. At the same time, describing the proportion of each type of incident is related to the main point or innovation of the article. Most of the data introduced in the research results show that the conclusions of this paper is different from those of previous studies, which plays a role in echoing the following.

Reviewer #2: Comment #5: L156-157: There are problems with the format of the manuscript. It is very necessary to check the language and format in the manuscript. Please be more rigorous about the manuscript.

*Authors' Response:* We have rigorously examined the format and statements of this section and optimized them accordingly. The language and format in the manuscript also received further scrutiny.
